# Screening of the Biocontrol Efficacy of Potent *Trichoderma* Strains against *Fusarium oxysporum* f.sp. *ciceri* and *Scelrotium rolfsii* Causing Wilt and Collar Rot in Chickpea

**DOI:** 10.3390/microorganisms12071280

**Published:** 2024-06-24

**Authors:** Ranjna Kumari, Vipul Kumar, Ananta Prasad Arukha, Muhammad Fazle Rabbee, Fuad Ameen, Bhupendra Koul

**Affiliations:** 1 Department of Botany, Lovely Professional University, Phagwara 144411, Punjab, India; ranjana20362@gmail.com; 2 Department of Plant Pathology, School of Agriculture, Lovely Professional University, Phagwara 144411, Punjab, India; vipul.19845@lpu.co.in; 3 Department of Nephrology and Hypertension, Mayo Medical Sciences, Rochester, MN 55902, USA; ananta.arukhaa@gmail.com; 4 Department of Biotechnology, Yeungnam University, Gyeongsan 38541, Republic of Korea; 5 Department of Botany and Microbiology, College of Science, King Saud University, Riyadh 11451, Saudi Arabia; fuadameen@ksu.edu.sa

**Keywords:** Biocontrol, *Trichoderma*, *Fusarium oxysporum*, *Sclerotium rolfsii*, Pathogens

## Abstract

Chickpeas contribute to half of the pulses produced in India and are an excellent source of protein, fibers, carbohydrates, minerals, and vitamins. However, the combination of the wilt and root rot diseases drastically lowers its yield. The use of antagonist microbes that restrict the growth of other phytopathogens is an ecofriendly approach to combat the serious threats raised by the plant pathogens. *Trichoderma* spp. are well known as biocontrol agents, especially against soil- and seed-borne phytopathogens. In this study, 21 *Trichoderma* isolates that were collected from different rhizospheric soils were evaluated against two notorious soil-borne pathogens, such as *Fusarium oxysproum* f.sp. *ciceri* and *Sclerotium rolfsii*. The maximum percentage of inhibition against the tested pathogens was observed in *Trichoderma* isolate PBT13 (72.97%, 61.1%) followed by PBT3 (72.23%, 59.3%). The mycelial extension rate method, dual culture (antagonism), production of cell-wall degrading enzymes (CWDs), and antifungal metabolites (by GC-MS) were used as selection criteria for potent *Trichoderma* isolates. Among the 21 isolates, PBT3, PBT4, PBT9, and PBT13 exhibited high antagonistic activity, production of antifungal metabolites, and chitinase and β-1,3-glucanase activity. These four species were subjected to molecular characterization using an internal transcribed spacer (ITS 1 and ITS4). The results of molecular characterization identified the four species as *T. virnes*, *T. asperellum*, *T. lixii,* and *T. harzianum*. Moreover, significant chitinase and β-1,3-glucanase activities of all *Trichoderma* isolates were recorded in the growth medium. *Trichoderma harzianum* (isolate PBT13) was found to exhibit the highest chitinase activity in terms of zone formation (4.40 ± 0.17 cm), whereas *Trichoderma virens* (isolate PBT3) exhibited the highest β-1,3-glucanase activity1.511 μmole/min. A GC-MS analysis of ethyl extracts from two isolates of *Trichoderma* (PBT9, PBT13) revealed the presence of 28 VOCs. Overall, this study suggests that these four *Trichoderma* strains are promising biological control agents (BCAs) and could be developed as bio-pesticides after stringent field trials for the management of soil-borne diseases of chickpeas.

## 1. Introduction

Chickpeas (*Cicer arietinum* L.) stand out as a crucial grain legume, renowned for their exceptional protein content [1]. With its globally recognized nutritional benefits and therapeutic potential, this crop holds significant agricultural value [2]. Moreover, it contributes to the enhancement of soil fertility by biologically fixing approximately 140 kg/ha of nitrogen [3]. Functioning as a substitute in cereal rotations and aiding soil replenishment, chickpea serves a vital role in farming systems as a fallow crop [4]. Since the 1990s, India has witnessed a notable surge in chickpea seed production, increasing from 4 million tons in 1990 to 9 million tons in 2019 [5]. This rise coincides with an expanding awareness of the health benefits associated with chickpea consumption and the growing adoption of plant-based diets. Consequently, there is a mounting demand for chickpea cultivation to bolster food security in developing nations [6]. Chickpeas hold the distinction of being the second most significant pulse crop, trailing only dry beans [1]. Its cultivation spans diverse regions, including tropical, subtropical, and temperate zones across South and West Asia, East and North Africa, Southern Europe, North and South America, and Australia [7]. In India, during the winter season, chickpeas are extensively cultivated across various states where they are grown, including Andhra Pradesh, Karnataka, Madhya Pradesh, Chhattisgarh, Uttar Pradesh, and Rajasthan [8]. Though they now only make up around half a million hectares of the roughly 12 million hectares of land used for chickpea production worldwide, they have significant economic potential in the sub-Saharan Africa (SSA) region [3].

Chickpea seeds serve as a significant source of nutrition for both humans and animals due to their abundant protein content, particularly lysine [9]. Moreover, the cultivation of chickpeas plays a crucial role in agricultural systems by substituting fallow periods in cereal rotations. This integration enhances production sustainability and diminishes the necessity for nitrogen fertilization by harnessing atmospheric nitrogen. Such characteristics underscore the importance of chickpea cultivation in bolstering food security in developing regions. However, the production of this crop is very low due to several constraints, such as biotic constraints (bacteria, fungi, viruses, nematodes, insects, rodents, anthropogenic activity, etc.) and abiotic constraints (temperature, water, salinity, light, heavy metal, pesticides, etc.) [10]. Among the pathogenic attacks, soil-borne pathogens like *Fusarium* and *Sclerotium rolfsii* attack chickpeas near the root and collar regions, thus producing symptoms including yellowing, which culminate in mortality within 25 to 30 days after sowing or wilting and drooping of leaves, as well as collar rot in plants [4]. It is the most vulnerable crop to several fungal, bacterial, and viral diseases [4,11,12]. Diseases and insect pests limit the yield of chickpeas. The central and peninsular regions of India are generally more affected by soil-borne diseases, such as Fusarium wilt, collar rot, and dry root rot, while northern, northern–western, and eastern India are more affected by foliar diseases, such as ascochyta blight and botrytis grey mold [13].

Collar rot is a recently identified soil-borne disease of chickpeas that can kill 55–95% of seedlings under ideal growing conditions, such as high soil temperature (25–30 °C) and significant rainfall [14]. Moreover, the management of these soil-borne diseases is very difficult for several reasons, including the wide host range of the pathogen, highly competitive saprophytic ability, and the ability to produce a resting structure (chlamydospore, sclerotium [15]. In recent times, wilt recurrence events in farmers’ fields have been increasing, and the severity of the issue is directly correlated with the increasing density of the pathogen inoculum in the soil [16].

Therefore, the management of these diseases should be carried out by reducing the amount and efficiency of the primary inoculum [4]. Due to this concern, strategies for managing soil-borne diseases in chickpeas often encompass a range of methods, including the use of partially resistant cultivars, early planting schedules, fungicide seed treatments, and biological approaches [17,18]. Currently, fungicide applications, both as seed treatments and as soil applications, stand out as the most effective means of preventing soil-borne illnesses in chickpeas [19]. However, this method may not be consistently effective due to several limitations, such as the diversity of soil-borne pathogens, human health hazards, detrimental effects on non-target/beneficial microorganisms, variations in environmental conditions, and the early development of fungicide resistance in soil-borne pathogens [20]. Chickpea resistance genes have recently been discovered, which protects it against Ascochyta blight, a disease caused by the fungal pathogen *Ascochyta rabiei* [11,21]; however, it is still hard to prevent a few soil-borne diseases like collar rot and dry root-rot of chickpea due to the lack of specific soil fungicides [22]. Over time, many previously resistant varieties have shown susceptibility due to the breakdown of their resistance mechanisms and the emergence of pathogen variability [23,24]. Rhizospheric competence is a critical component of an effective biocontrol agent (beneficial microorganisms and their products). The different soils, crops, and pathogens affect the microbial population in the rhizospheric zone [25]. Thus, understanding the significance of rhizospheric microorganism for controlling soil-borne diseases is a promising research field, as seen by numerous reports published during the period from 2000 to 2019 [26,27]. Certain rhizosphere and endophytic microorganisms mostly include antagonistic fungi *Trichoderma* spp. and bacterial genera, *Bacillus*, *Pseudomonas,* and *Streptomyces*, which act as potential candidates due to their mutualistic interactions with the plants and rhizospheric soils by exchanging nutrients and protecting the plants from their pathogens by producing several CWDs enzymes [28]. These microbes coexist with the host plant in close proximity without causing any detrimental effect [29]. Rhizospheric and endophytic microbes have been used in conjunction in a number of experiments to promote plant growth and inhibit illness. For example, Sundaramoorthy et al. (2012) reported the synergistic interactions between rhizospheric *Bacillus subtilis* and endophytic *Pseudomonas fluorescens* for suppressing *Fusarium solani* causing wilt disease and promoting the growth of chili [30]. Moreover, Emami et al. (2019) showed the synergistic effect of endophytic and rhizospheric bacteria in increasing wheat growth [31].

The use of biocontrol agents like *Trichoderma* spp., *Pseudomonas* spp., *Bacillus* spp., and Mycorrhizal fungus may be a viable alternative to effectively control plant diseases, which also reduces the dependency on chemicals [32]. *Trichoderma*, a biocontrol agent, mostly survives in the soil, plant surface, and other ecological conditions and is commonly used for several plant diseases [32,33]. In order to safeguard crops around the world, alternative management strategies, specifically fungal biological control agents, or BCAs, have gained much attention in recent years. As of now, antagonistic fungi are primarily recognized for their potential to reduce the inoculum density of pathogenic fungi [34]. Among these, fungi belonging to the genus *Trichoderma* are employed successfully as biocontrol agents (BCAs) as well as biopesticides all over the world [35,36].

*Trichoderma* is generally applied in the field, especially to control soil-borne diseases in different crops [37]. For the development of potential biopesticides against plant diseases, he screening of *Trichoderma* isolates is required [38]. Certain strains of *Trichoderma* possess potent biocontrol properties against *Fusarium oxysporum* f.sp. ciceri (FOC) and *Sclerotium rolfsii* (SR), thereby exhibiting efficacy in reducing wilt and collar rot incidence in chickpea plants. This efficacy may vary depending on the specific *Trichoderma* strains screened, their modes of action, and their interactions with the pathogens and the chickpea plants.

The objectives of this research were (i) the isolation of *Trichoderma* from 21 soil samples of different districts of Punjab; (ii) the molecular characterization of *Trichoderma* spp.; (iii) the comparative assessment of the chitinase and β-1,3-glucanase activities, growth and antagonistic activity of *Trichoderma* isolates against FOC and SR and the identification of potential candidate(s) based on their bio-efficacy for a pilot-scale-experiment; and (iv) the identification of secondary metabolites from potential strain(s). Thus, it shall open new vistas for *Trichoderma*-based biopesticides for the management of the aforementioned pests in field conditions.

## 2. Materials and Methods

### 2.1. Pathogenic Strains

Soilborne pathogens, such as *Fusarium oxysporum* f.sp. *ciceri* (FOC) and *Sclerotium rolfsii* (SR), were used in this research, procured from the Indian-type culture collection (ITCC), New Delhi.

### 2.2. Collection of Rhizospheric Soil Samples

Twenty-one *Trichoderma* isolates were isolated from uncultivated soil in different districts of Punjab (Table 1) and screened for their biocontrol efficacy against the aforementioned soil-borne pathogens. The soil samples were aseptically collected and subsequently preserved at 4 °C in a freezer to maintain the viability of microorganisms during transportation and storage and to ensure the availability of these soil samples for the isolation of *Trichoderma* and further investigation.

### 2.3. Isolation of Trichoderma *sp.*

The rhizospheric soil samples were processed by using the serial dilution technique for the isolation of *Trichoderma* [39]. The samples were prepared in sterilized distilled water and then serially diluted up to five-fold (10^−5^). The 0.5 mL diluted sample was inoculated on the surface of the selective medium, Rose Bengal medium (RBA), using a glass spreader [40]. The inoculated plates were incubated in a BOD incubator (Navyug, India) at 28 °C for three days. To obtain a pure isolate of *Trichoderma*, green colonies were selected and cultured on potato dextrose agar (PDA) medium, and putative *Trichoderma* colonies were purified by using the single-spore isolation technique [41]. The purified cultures were observed for cultural and morphological characteristics. The microscopic characteristics, including color, shape of conidia, and philaids, of the isolates were examined under a light microscope, and the isolated *Trichoderma* was confirmed at the genus level [42,43]. All purified strains were maintained on PDA slants at 4 °C for further analysis [42].

### 2.4. Molecular Characterization of Trichoderma *sp.*

The morphologically identified *Trichoderma* isolates that showed more than 60% and 70% inhibition against phytopathogens *Sclerotium rolfsii* and *Fusarium oxysporum*, respectively, were subjected to Sanger sequencing method the internal transcribed spacer (ITS) region of genomic DNA using universal primers ITS1 and ITS4 [44]. For this purpose, the cetyltrimethylammonium bromide (CTAB) method was used to extract the whole genomic DNA of four isolates of *Trichoderma* [44]. The ITS1 and ITS4 primers were used to amplify the internal transcribed spacer (ITS) region of the fungal genomic DNA by thermal cycler (Applied biosystem, USA) [44]. The following parameters were used for the PCR: a 3 min initial denaturation at 94 °C, 30 cycles at 94 °C for 15 s, 50 °C for 1 min, 72 °C for 45 s, and a final extension at 72 °C for 7 min. A 50 µL PCR reaction mixture were prepared using the following components: 1× PCR buffer (GeNeI Bengaluru, Karnataka, India), 200 µM dNTP’s (GeNeI Bengaluru, Karnataka) 1.5 mM MgCl_2_, 5 pmol of each primer, 10 ng of gDNA, and 1.5 units of Taq DNA polymerase (NEB, USA). The ITS region of PCR amplified products (600 to 650 bp) was sequenced by Mr. Biologist India Pvt. Ltd. (Pune, India) through Sanger sequence technology. Sequences of *Trichoderma* were obtained from the Genbank database and utilized to infer the relationship, and these are used for phylogenetic analysis. *Trichoderma* species were identified using the highest identity, maximum query 100% [45]. MEGA software, version 11.0 was used to construct a phylogenetic tree, and finally, the sequences were deposited in GenBank.

### 2.5. Biocontrol of Trichoderma Isolates on FOC and SR Pathogens

The impact of *Trichoderma* on the mycelial growth of FOC and SR was assessed through four in vitro assays: (i) growth rate, (ii) a dual culture assay to evaluate the antagonistic effect against pathogen, (iii) the production of lytic enzymes (chitniase, β-1,3-glucanase), and (iv) the production of secondary metabolites. All in vitro assays were performed in a completely randomized design with three replicates and each experiment was repeated twice.

#### 2.5.1. Evaluation of Growth Rate of *Trichoderma* Isolates/Pathogens

The mycelial extension rate method was used to evaluate the growth rate of fungi. Five-day-old mycelium discs were placed at the periphery of Petri plates (9 cm) containing PDA (one disc per plate) and incubated at 25 ± 2 °C (12/12 light/dark photoperiod) for 96 h. During the incubation period, *Trichoderma* growth was quantified by measuring the distance from the center of the mycelium disk to the outermost edge of the mycelium growth [46]. The growth rate test was performed by calculating the growth rate (mm h^−1^) using the following formula:

$$Mycelium\;growth\;rate=\frac{C2-C1}{T2-T1}$$

where C2 = *Trichoderma* growth after 96 h; C1 = *Trichoderma* growth after 24 h; T2 = Pathogen growth after 96 h and T1 = Pathogen growth rate after 24 h.

#### 2.5.2. Dual Culture Assay

A dual culture method was used to evaluate the antagonistic behavior of *Trichoderma* isolates against chickpea wilt and collar rot pathogens FOC (ITCC no. 6341) and SR (ITCC no. 8527) [34]. A sterile cork borer was used to cut discs on plates containing the culture of *Trichoderma* and pathogens, respectively, which were kept on PDA plates opposite to each other from the edges and incubated at 28 ± 2 °C for 96 h. As a control, another Petri plate was inoculated with pathogens only. The inhibition percentage of pathogens was measured using the following equation [47].

$$Inhibition\;percentage\;(I)=\frac{Control-Treatments}{Control}\times 100$$

Four promising *Trichoderma* species [*T. harzianum* (accession no.: MF87546.1)], *T. virens* (accession no.: MN452840.1), *T. asperellum* (accession no.: MN046976.1), and *T. lixii* (accession no.: MK288146.1)] were selected from among the twenty-one aforementioned isolates on the basis of their growth rate and antagonistic activity against FOC and SR. These were then assessed for multifarious activities, (i) genome sequencing through Sanger technology, and (ii) GC-MS for the identification of fungal metabolites.

#### 2.5.3. Cell Wall Degradation Enzymatic Assay

Five-day-old 5 mm mycelial discs of *Trichoderma* isolates that were cultured on MYPG medium (malt extract + yeast extract + glucose) were inoculated into trace element solution (TLE medium). Lyophilized pathogen cell walls (@5% *S. rolfsii* and FOC) were utilized as nutrient sources (carbon and nitrogen), respectively. The culture was grown in a 150 mL conical flask at 28 °C kept on a shaker set at 120 rpm. After 48 h of continuous shaking, the mycelium was collected by passing it through Whatman filter sheet, and the filtrate was subsequently used as a source of enzymes.

#### 2.5.4. β-1,3-Glucanase Assay

Laminarin (0.75% *w*/*v*) was employed as a substrate in sodium acetate buffer (50 mM, pH 5.0) to quantify the β-1,3-glucanase activity of *Trichoderma* [46]. For ten min, the assay mixture (10 μL of enzyme solution and 20 μL of laminarin solution) was incubated at 50 °C in a water bath. Following the incubation, 100 μL of 3,5-dinitrosalicylic acid (DNS) was added, and the reaction mixture was allowed to incubate for 5 min at 95 °C. The reducing sugar content was examined spectrophotometrically at 540 nm. The amount of β-1,3-glucanase that could catalyze the release of 1 μmol of reducing sugar equivalents per minute was defined as one unit.

#### 2.5.5. Chitinase Assay

Colloidal chitin was prepared using a modified version of Roberts and Selitrennikoff’s (1988) procedure using commercial chitin. In order to hydrolyze chitin, 40 g of powdered chitin was gradually combined with 500 mL of concentrated HCl and left on a magnetic stirrer at 4 °C (refrigerator) for the entire night. Following an overnight hydrolysis process, colloidal chitin was extracted using 95% cooled ethanol and left overnight to neutralize the ethanol. After that, it was centrifuged for 20 min at 3000 rpm. Centrifugation at 3000 rpm for 5 min at 4 °C was carried out to wash the pellets with distilled water in order to remove any remaining alcohol. With 90–95% moisture content and a soft, pasty consistency, the resulting colloidal chitin was kept at 4 °C until needed again.

Chitinase detection medium consisted of magnesium sulfate (0.3 g), ammonium sulfate (3.0 g), monopotassium phosphate (2.0 g), citric acid monohydrate (1.0 g), agar–agar (15 g), Tween-80 (200 μL), colloidal chitin (4.5 g), and bromocresol purple (0.15 g). After the pH was adjusted to 4.7, the media were autoclaved at 121 °C for 15 min. Lukewarm medium was poured into Petri plates and allowed to solidify under laminar flow. A seven-day-old culture of *Trichoderma* (7 mm in diameter) was inoculated in the center of each Petri plate, incubated in a BOD incubator (Meta-Lab, India) at 28 ± 2 °C, and observed for a period of 7 days for colored zone formation.

#### 2.5.6. Extraction and Identification of Secondary Metabolites

To prepare the crude extract of *Trichoderma*, two isolates were inoculated into two different conical flasks (250 mL) containing 100 mL of PDB separately and kept in a BOD incubator for nine days at 28 ± 2 °C. After nine days of incubation, to avoid mycelium, hypha, and other fragments, cultures were filtered using Whatman filter paper to obtain the crude extract of the secondary metabolites. To check the absence of conidia and mycelia (to ensure optimal filtration), a control assay was performed by inoculating 30 µL of the final filtrate on a PDA containing Petri dishes. Inoculated Petri dishes were examined for *Trichoderma* growth.

The solvent extraction method was used to extract secondary metabolites, and ethyl acetate and culture filtrate were combined in a 1:1 ratio. The upper solvent layer containing compounds was separated from the aqueous layer (PDB medium) using a separating funnel. A vacuum rotary evaporator (Aditya scientific Technologies, India) was used to remove ethyl acetate from the filtrate. It was set up to operate at 40 °C and 70 rpm until the extract was decreased to 4 mL.

Thereafter, it was kept for further examination in a deep freezer (−20 °C). Gas chromatography mass spectroscopy (GC-MS) (Thermo Scientific, USA) was utilized to examine the ethyl acetate extracts. The GC-MS analysis was performed using Thermo Scientific-Ceres 800 g and MS DSQ II, and the silica column was packed using Elite-5MS (5% biphenyl, 95% dimethylpolysiloxane, 30 m × 0.25 mm ID × 250 μmdf). For the component separation process, helium gas was employed as a carrier gas, with a steady flow rate of 1 mL per min. The temperature of the injector was maintained at 260 °C throughout the chromatographic run. The apparatus was calibrated to measure 1 mL of extract at 60 °C for 2 min, then 300 °C at a rate of 10 °C/min, and 300 °C for 6 min. The mass detector was operated with the following specifications: an electron impact in the ionization mode at 70 eV, an ion source and transfer line temperature of 230 °C, a 0.2 s scan period, and a 0.1 s scan interval. The component spectra were compared to those stored in the GC-MS NIST library-2008.

#### 2.5.7. Statistical Analysis

A two-way ANOVA test was conducted using SPSS software version 22 to analyze the data. Duncan’s multiple range test (DMRT) was employed to separate the means at a 5% significance level. The GC-MS analysis was performed using Mega-11 version 11.0.13 software.

## 3. Results

### 3.1. Isolation of Trichoderma from the Rhizosphere

Twenty-one isolates of *Trichoderma* were procured from different districts of Punjab. Initially, colonies were white, including white–bright-red, white–bright-purple, white–bright-yellow, and completely white. After 48 h of incubation on RBA, these colonies turned green. Thereafter, these colonies were transferred on PDA plates wherein the mycelia spread quickly and formed sponge-like structures as well as concentric rings. All the *Trichoderma* isolates produced conidiophores (long and thick) with branches. The apex of each branch resembled a little bottle (known as phialides) with oval-shaped conidia. Some isolates developed different morphologies on the PDA Petri plate like light and dark green/yellowish colonies. The *Trichoderma* growth rate was evaluated with respect to FOC and SR.

**Figure 1:**
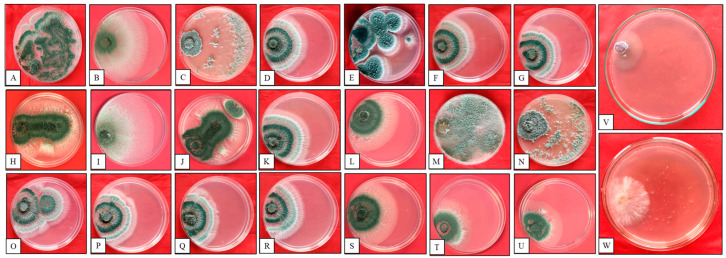
Growth rate of *Trichoderma* isolates on PDA media. Plates from **A**–**U** refer to the *Trichoderma* isolates PBT1 to PBT21, respectively. **V** and **W** are control for FOC and SR respectively.

The growth rate of *Trichoderma* isolates exhibited considerable variability (1.02–2.61 cm and 0.6–1.6 cm) between isolates after 96 h of incubation with respect to FOC and SR, respectively. Notably, all the *Trichoderma* isolates demonstrated accelerated growth compared to the tested phytopathogens, as shown in Table 1 and Figure 1. Out of 21 isolates, PBT13 exhibited the highest growth rate (2.61 cm), followed by PBT3 (2.53 cm), PBT9 (2.51 cm), and PBT4 (2.22 cm). The investigation revealed that PBT5 exhibited the lowest growth rate among the tested *Trichoderma* isolates, with a measured value of 1.02 cm, in comparison to FOC, while with SR, PBT13 showed the highest growth rate of 1.6 cm, followed by PBT3 (1.5 cm), PBT9 (1.5 cm), and PBT4 (1.3 cm). The lowest growth rate was observed in isolate PBT21 (0.6 cm). Thus, the growth rate of PBT13 was found to be superior with respect to both the pathogens.

#### 3.1.1. Antagonistic Activity of *Trichoderma* Isolates against FOC and SR

**Figure 2:**
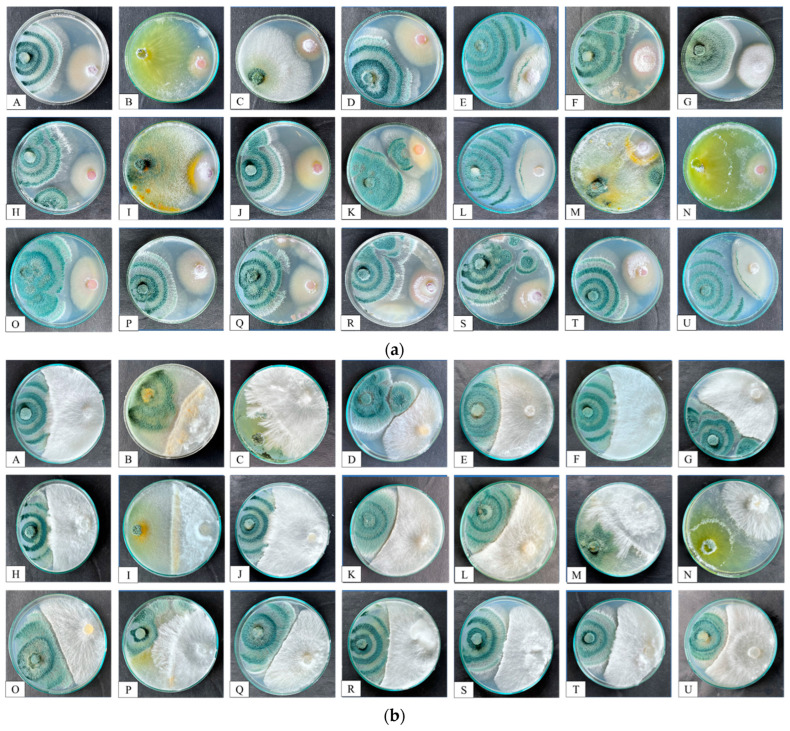
(**a**) Antagonistic activity of *Trichoderma* isolates against soil-borne pathogen *Fusarium oxysporum* f.sp. *ciceri* (FOC). Plates from A–U refer to the *Trichoderma* isolates PBT1 to PBT21, respectively. (**b**) Antagonistic activity of *Trichoderma* isolates against soil-borne pathogens *Sclerotium rolfsii* (SR). Plates from A–U refer to the *Trichoderma* isolates PBT1 to PBT21, respectively.

Results from the antagonistic activity revealed that most of the isolates suppressed the mycelium growth of FOC and SR >58% and 45%, respectively, as shown in (Table 1 and Figure 2a,b). Isolates PBT13, PBT3, PBT9, and PBT4 were found to be superior as they inhibited mycelial growth of both FOC and SR. Among the 21 isolates, only 5 (PBT1, PBT6, PBT8, PBT16 and PBT 17) inhibited the growth of the SR pathogen by less than 45%, whereas, in the case of FOC, none of the *Trichoderma* isolates inhibited the mycelium growth by less than 50%. The overall mycelial growth inhibition percentage of both pathogens by *Trichoderma* isolates were recorded between 36.3 and 72.97%.

#### 3.1.2. Estimation of Chitinase and β-1,3-Glucanase Activities

Enzymes that break down cell walls (CWDs), such as chitinase and β-1,3-glucanase, are essential for *Trichoderma* species’ ability to oppose phytopathogens. There is a positive correlation between the synthesis of extracellular enzymes and the antagonistic activity of *Trichoderma* isolates. A faster, more sensitive, more affordable, and reliable CWD assay is widely desired because there is a continuous quest for new isolates of *Trichoderma* spp. to be employed in biocontrol regimes.

**Figure 3:**
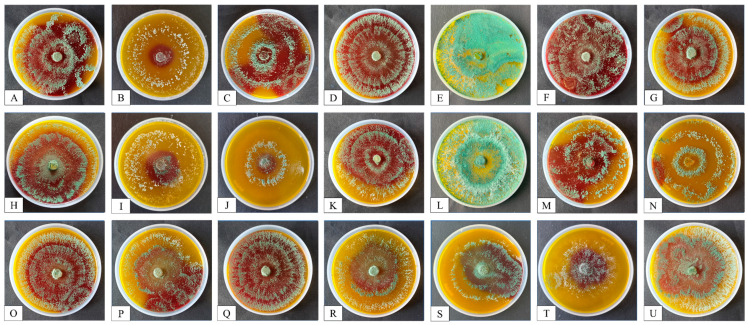
Screening of *Trichoderma* isolates for chitinase activity using a medium enriched with colloidal chitin. Plates from **A**–**U** refer to the *Trichoderma* isolates PBT1 to PBT21, respectively.

The diameter of the purple-colored zone, which was divided into four groups (1) = no chitinase activity, (2) = low chitinase activity, (3) = medium chitinase activity, and (4) = high chitinase activity was used to measure the chitinase activity of *Trichoderma* isolates on the colloidal-chitin-supplemented medium (Table 2 and Figure 3).

Among the 21 *Trichoderma* isolates, only 6 isolates (PBT1, PBT3, PBT4, PBT11, PBT13, and PBT14) showed the highest chitinase activity. Eight isolates (PBT2, PBT9, PBT10, PBT16, PBT17, PBT18, PBT19 and PBT20) and five isolates (PBT6, PBT7, PBT8, PBT15, PBT21) exhibited low and medium chitinase activity, respectively. Only two isolates (PBT5 and PBT12) did not exhibit chitinase activity.

#### 3.1.3. β-1,3-Glucanase Activity

To estimate the amount of β-1,3-glucanase, standard curves were plotted using glucose. To accurately measure amounts of enzyme in the *Trichoderma* isolates, a range of concentrations (10, 20, 30, 40, 50, 60, 70, 80, 90, and 100 mg mL^−1^) of glucose was used. The enzyme activity values observed using the standard curve are shown in Table 2.

By cultivating each *Trichoderma* isolate on a medium supplemented with 1% laminarin as a carbon source, the β-1,3-glucanase activity of each isolate was evaluated. The outcomes demonstrated that the β-1,3-glucanase activity of these isolates varied widely (Table 2). The highest β-1,3-glucanase activity (1.511 μmole min^−1^) was demonstrated by isolates PBT13 and PBT3, which were followed by PBT4 (1.442 μmole min^−1^) and PBT14 (1.488 μmole min^−1^). PBT17 displayed the lowest β-1,3-glucanase activity (0.435 μmole min^−1^).

#### 3.1.4. Molecular Identification of *Trichoderma* Isolates

**Figure 4:**
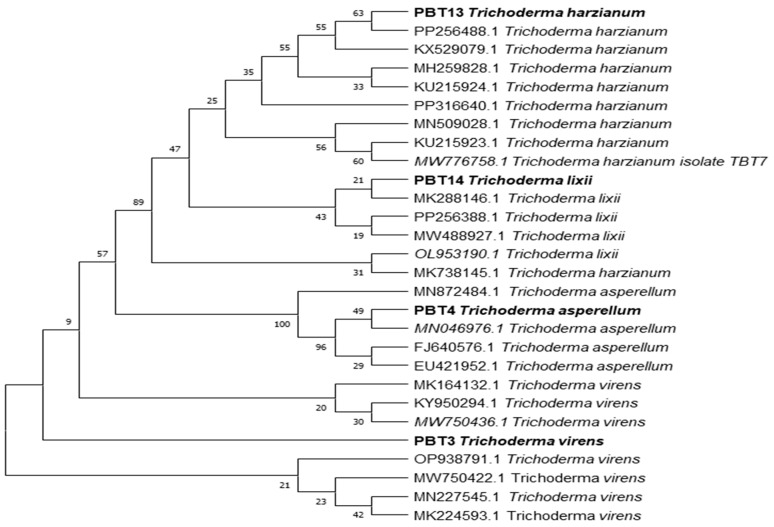
Phylogenetic tree construction of *Trichoderma* strains using MEGA 7.1 software.

**Figure 5:**
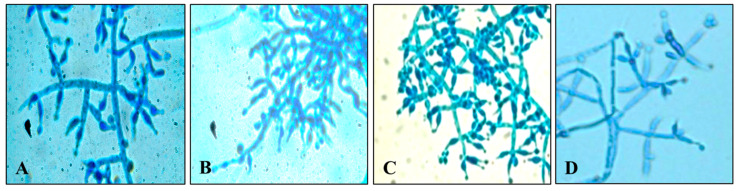
Microscopic images of identified *Trichoderma* species. (**A**) *T. virens*; (**B**) *T. asperellum*; (**C**) *T. harzianum*; and (**D**) *Trichoderma lixii*.

The molecular identification of the *Trichoderma* isolates was performed using internal transcribed spacer (ITS) sections of their rDNA. Primers that are universal were used to amplify the ITS regions (ITS 1 and ITS 4). The amplified products (between 500 and 600 bp) were sent to Mr. Biologist in Pune, India, for partial genome sequencing. The acquired sequences were uploaded to the online database of the National Centre for Biotechnology Information (NCBI) for verification. Table 3 displays the complete similarity of the *Trichoderma* isolates *T. harzianum*, *T. asperellum*, *T. virens*, and *T. lixii* with the strains currently listed on the NCBI. A phylogenetic tree (Figure 4) based on the sequence analysis of the ITS sections was created using the neighbor-joining method in the MEGA 7.1 program to clarify the genetic relatedness between the *Trichoderma* isolates. Based on the ITS sequences, the phylogenetic analysis of four *Trichoderma* stains revealed great proximity (with 100% bootstrap support) between *T. virens*, *T. asperellum*, *T. harzianum*, and *T. lixii*. These species also showed a close connection with other strains within the same genus (Figure 4). The microscopic images of these species are shown in Figure 5.

#### 3.1.5. GC-MS Analysis

**Figure 6:**
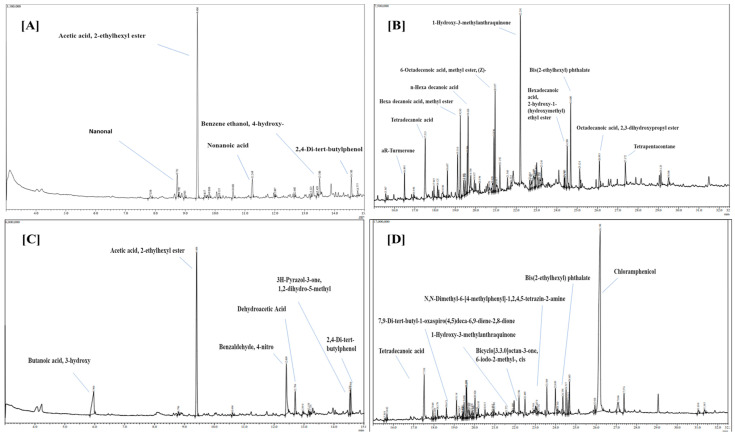
Gas Chromatography-Mass Spectroscopy (GC-MS) of crude extracts of (**A**) *Trichoderma harzianum* (RT: 3–15 min), (**B**) (RT: 15–33 min), and (**C**) *Trichoderma lixii*. (RT: 3–15 min), (**D**) (RT: 15–33 min). RT = Retention time.

The ethyl acetate extracts of *T. harzianum* and *T. lixii* showed thirty-eight antifungal compounds (through GC-MS analysis), as shown by the peaks in GC-MS chromatogram (Figure 6). Table 4 displays a list of antifungal compounds together with their retention time (RT), molecular weight, peak area and molecular formulae. Typical chromatograms and mass spectra of the compounds identified from the ethyl acetate extracts of *T*. *harzianum* and *T*. *lixii* are shown, respectively, in Figure 6. The identified compounds with sharp peaks and maximum peak area from these species were acetic acid, 2-ethylhexyl ester, tetradecanoic acid, n-Hexadecanoic acid, 6-Octadecenoic acid, methyl ester, (Z)-, 1-Hydroxy-3-methylanthraquinone, and chloramphenicol.

## 4. Discussion

Rapid growth is one of the characteristic features of *Trichoderma* that is crucial for competition for food and space in pathogenic fungi. In our study, we found that *Trichoderma* grows faster than phytopathogens. It was clearly observed that *Trichoderma* occupied more space of the Petri plate than the pathogenic fungi, which showed obvious competition. However, *T. harzianum*’s slower growth rate indicates that it will be more effective if it is planted in fields prior to the pathogen’s arrival.

### 4.1. Antagonistic Activity

Dual culture interaction between *Trichoderma* and two phytopathogenic fungi *F. oxysporum* f.sp. *ciceri* and *S. rolfsii* showed that the *Trichoderma* isolate reduces the growth of mycelium of the pathogens [48]. The data shown in Figure 2a,b revealed that the efficacy rate of *Trichoderma* isolates is different in inhibiting the mycelium growth of *F. oxysporum* f.sp. *ciceri* and *S. rolfsii*. The most effective *Trichoderma* isolate was found to be PBT13 (*T. harzianum*) followed by isolates PBT3 (*T. virens*), PBT9 (*T. lixii*), and PBT4 (*T. asperellum*) against the FOC and SR. The least effective isolates were PBT5 and PBT15 in FOC and PBT1 and PBT16 in SR. Paul et al. (2021) revealed that among the tested *Trichoderma* isolates, *T. harzianum* was found to be the most potential biocontrol agent, as evidenced by suppression of the mycelial growth of phytopathogens [49]. The results are in consonance with those of Qualhato et al. (2013) with *T. harzianum* against phytopathogens [50]. The possible biocontrol mechanism (mycoparasitism) among both *Trichoderma* and phytopathogens was hyphal interaction, as well as the production of CWDs such as chitinase and β-1,3-Glucanase, which led to the breakdown of the pathogens’ cell walls.

### 4.2. Enzyme Activity (Chitinase and β-1,3-Glucanase)

Various CWDs, including β-1,3-glucanase and chitinase, are produced by *Trichoderma* isolates and are essential to *Trichoderma*’s antagonistic activity. According to Saravanakumar et al. (2016), there is a positive link between the antagonistic capacity of *Trichoderma* isolates against phytopathogens and their production of CWD enzymes. It was found that 10 isolates of *Trichoderma* that had significant biocontrol ability against pathogens were also capable of secreting β-1,3-glucanase and chitinase [51]. Our findings are consistent with past findings [52]. The PBT13 isolate exhibited the highest β-1,3-glucanase enzyme activity, followed by PBT3, PBT14, and PBT1. In addition to competing with pathogens for nutrients and space, *Trichoderma* can also break down their cell walls, distort or even digest their hyphae, and stop them from growing. Additionally, it has been shown that *Trichoderma* CWDs such as chitinase and glucanase are essential to the pathogen-induced hyperparasitism process.

### 4.3. Molecular Identification of Trichoderma Isolates

Previous studies have shown that the ITS region is the universal barcode that has been used for accurate identification of *Trichoderma* isolates [43,53]. In the current study, four *Trichoderma* strains were identified using ITS-primers (ITS1 and ITS4) and identified as *T. harzianum*, *T. asperellum*, *T. virens*, and *T. lixii*. These strains were further confirmed by online database searches (NCBI), which were carried out with BLAST and revealed a 100% similarity index. The current study revealed that the amplification of a PCR produces approximately 600 base pairs in size from the ITS region of the *Trichoderma* spp. Similar findings have been shown by several researchers [43,54].

### 4.4. GCMS Analysis

GC-MS analysis showed that biocontrol agents like *Trichoderma* produced various volatile organic compounds (VOCs), including alcohols, acids, esters, sesquiterpenes, and ketones. These compounds provide specific benefits in various biological mechanisms, like competition, symbiosis, metal transportation, growth differentiation, signaling, and mycoparasitic behavior [55], and also show antifungal and antibacterial activities against various phytopathogens like *Fusarium* spp. and *Sclerotium* spp. [56].

As mentioned above, the crude extracts of *T. harzianum* and *T. lixii* showed 15 and 13 antifungal and antipathogenic compounds, respectively. Stracquadanio et al. (2020) has shown the antimicrobial properties of bioactive compounds synthesized by two *Trichoderma* sp., such as *T*. *asperellum* and *T*. *atroviride* in liquid medium [57]. Oviya et al. (2022) also explained that hexadecenoic acid has antibacterial and antifungal activity against plant pathogenic fungi *Fusarium oxysporum* [58]. Shilov et al. (2022) also explained that 3-hydroxy-butanoic acid has antibacterial effects against the plant pathogenic fungi [59]. Moreover, Habib et al. (2009) also explained that acetic acid, 2-ethylhexyl ester has an antibacterial effect against the plant pathogenic fungi [60]. Our study also provided evidence that *Trichoderma* spp. emits volatile organic compounds (VOCs) that have antifungal effects against soil-borne pathogens and could be used as the best substitute for synthetic fungicides. Biofumigation by *Trichoderma* is an eco-friendly and less hazardous alternative way to manage postharvest diseases.

## 5. Conclusions

The aim of this experiment was to identify a robust strain of *Trichoderma* and to assess its ability to biocontrol two virulent phytopathogens, *F*. *oxysporum* f.sp. *ciceri* and *S*. *rolfsii*. *T. virens*, *T. asperellum*, *T. harzianum*, and *T. lixii* were identified as promising candidates for biocontrol agents against soil-borne diseases, based on the results of the in vitro screenings and their strong ability to suppress the growth of both pathogens. Our results indicate that the application of potent *Trichoderma* isolates could provide an alternative and sustainable disease management strategy for controlling soil-borne pathogens. In the future, a consortium of these potent candidate strains could be evaluated, and their hidden metabolites along with synergism/efficacy should be studied on other soil-borne and oomycete classes of pathogens. Thus, further studies are required on (i) the pilot and mass-scale production of these promising strains; (ii) the efficacy of the formulation and compatibility with different carrier materials; (iii) increasing the shelf life of formulation; (iv) the curative effect of the formulation in field conditions; and (v) the synergistic effect of the strains, before commercialization. Furthermore, more research should be performed in the field to determine the bioefficacy of these *Trichoderma* isolates in preventing soil-borne infections from proliferating and inhibiting their resting phases (sclerotia and chlamydospore).

Our research contributes significantly to the understanding of *Trichoderma* as a biocontrol agent. The identification of potent *Trichoderma* isolates that suppress the growth of virulent phytopathogens highlights their potential in sustainable agriculture. The correlation between enzyme production and antagonistic activity underscores the importance of CWDs enzymes in biocontrol mechanisms. Additionally, the identification and analysis of VOCs produced by *Trichoderma* offer new insights into alternative, eco-friendly pest-management strategies. These findings pave the way for future research on the mass production, formulation development, and field application of these biocontrol agents, ultimately contributing to more sustainable and effective agricultural practices.

## Figures and Tables

**Table 1:** Evaluation of growth rate and antagonistic activity of *Trichoderma* isolates against FOC and SR.

Sr. no.	Isolates	Location	Growth with Respect to FOC (cm)	Growth Rate with Respect to SR (cm)	Percentage Inhibition of FOC	Percentage Inhibition of SR
1.	PBT1	Faridkot	1.55 ± 0.15 ^bc^	0.9 ± 0.09 ^bc^	68.52 ± 8.48 ^bc^	38.9 ± 5.56 ^ik^
2.	PBT2	Barnala	1.30 ± 0.74 ^d^	0.8 ± 0.42 ^c^	61.12 ± 5.56 ^cd^	51.9 ± 6.41 ^cdef^
3.	PBT3	Bathinda	2.53 ± 0.13 ^ab^	1.5 ± 0.03 ^a^	72.23 ± 5.56 ^b^	59.3 ± 6.41 ^bc^
4.	PBT4	Amritsar	2.22 ± 0.56 ^abc^	1.3 ± 0.33 ^ab^	70.00 ± 4.00 ^bc^	55.6 ± 5.56 ^bcd^
5.	PBT5	Fatehpur Sahib	1.02 ± 0.70 ^c^	0.6 ± 0.34 ^c^	58.52 ± 2.80 ^d^	45.6 ± 4.00 ^efghi^
6.	PBT6	Fazilka	1.63 ± 0.09 ^cd^	0.9 ± 0.05 ^bc^	69.26 ± 2.80 ^bc^	41.1 ± 2.23 ^hij^
7.	PBT7	Ferozpur	1.35 ± 0.09 ^d^	0.8 ± 0.05 ^c^	62.60 ± 3.40 ^cd^	49.3 ± 5.70 ^defg^
8.	PBT8	Gurdaspur	1.20 ± 0.56 ^d^	0.7 ± 0.33 ^c^	61.12 ± 5.56 ^cd^	44.1 ± 1.70 ^fghij^
9.	PBT9	Malerkotla	2.51 ± 0.12 ^ab^	1.5 ± 0.00 ^a^	72.23 ± 5.56 ^b^	55.9 ± 1.70 ^bcd^
10.	PBT10	J&K	1.24 ± 0.43 ^d^	0.7 ± 0.24 ^c^	60.75 ± 6.12 ^cd^	47.0 ± 2.79 ^efgh^
11.	PBT11	Jalandhar	1.64 ± 0.11 ^cd^	1.0 ± 0.07 ^bc^	62.23 ± 4.01 ^cd^	45.2 ± 1.28 ^efghi^
12.	PBT12	Kapurthala	1.33 ± 0.28 ^d^	0.8 ± 0.16 ^c^	57.41 ± 1.70 ^d^	52.6 ± 2.80 ^cde^
13.	PBT13	Ludhiana	2.61 ± 0.06 ^a^	1.6 ± 0.05 ^a^	72.97 ± 5.01 ^b^	61.1 ± 5.56 ^b^
14.	PBT14	Himachal Pradesh	1.60 ± 0.85 ^cd^	0.9 ± 0.49 ^bc^	69.26 ± 2.80 ^bc^	53.0 ± 2.80 ^cde^
15.	PBT15	Mansa	1.75 ± 0.43 ^bcd^	1.0 ± 0.25 ^bc^	58.15 ± 2.80 ^d^	50.0 ± 5.56 ^def^
16.	PBT16	Moga	1.57 ± 0.12 ^cd^	0.9 ± 0.07 ^bc^	66.67 ± 2.22 ^bcd^	36.3 ± 2.79 ^j^
17.	PBT17	Muktsar	1.52 ± 0.09 ^cd^	0.9 ± 0.05 ^bc^	66.30 ± 2.79 ^bcd^	41.9 ± 2.80 ^ghij^
18.	PBT18	Nawanshahr	1.57 ± 0.14 ^cd^	0.9 ± 0.09 ^bc^	61.49 ± 7.14 ^cd^	50.4 ± 5.01 ^def^
19.	PBT19	Patiala	1.05 ± 0.16 ^d^	0.6 ± 0.09 ^c^	61.12 ± 5.56 ^cd^	50.4 ± 5.01 ^def^
20.	PBT20	Sangrur	1.49 ± 1.08 ^cd^	0.8 ± 0.49 ^c^	65.93 ± 5.60 ^bcd^	46.3 ± 3.39 ^efghi^
21.	PBT21	Tarn Taran	1.11 ± 0.28 ^d^	0.6 ± 0.16 ^c^	61.12 ± 5.56 ^cd^	49.3 ± 4.49 ^defg^
	Control		-	-	90.00 ± 0	90.0 ± 0
	CD		0.737	0.397	7.812	6.930
	SE(d)		0.364	0.196	3.863	3.427

DMRT test was used to check the significance level among the value. Values in a column with different lowercase letters are significantly different; *p* ≤ 0.00001). FOC: *Fusarium oxysporum* f.sp. cicero; SR: *Sclerotium rolfsii*.

**Table 2:** β-1,3-Glucanase and chitinase activity of different *Trichoderma* isolates.

S.no.	Isolates	β-1,3-Glucanase Activity (μmole min^−1^)	Chitinase Activity/Zone Formation (cm)
1	PBT1	1.407	4.00 ± 0.50 ^abcd^
2	PBT2	0.828	2.17 ± 0.76 ^fgh^
3	PBT3	1.511	4.33 ± 0.29 ^ab^
4	PBT4	1.442	4.23 ± 0.25 ^abc^
5	PBT5	1.048	0.00 ± 0.00 ^h^
6	PBT6	1.349	3.67 ± 0.7 6 ^abcde^
7	PBT7	1.025	3.33 ± 0.29 ^abcdefg^
8	PBT8	1.28	3.17 ± 1.15 ^abcdefg^
9	PBT9	1.488	4.33 ± 0.29 ^ab^
10	PBT10	0.898	2.67 ± 2.08 ^defgh^
11	PBT11	0.666	4.03 ± 0.06 ^abc^
12	PBT12	1.187	0.00 ± 0.00 ^i^
13	PBT13	1.511	4.40 ± 0.17 ^a^
14	PBT14	0.504	1.47 ± 0.00 ^h^
15	PBT15	1.245	3.97 ± 0.25 ^abcd^
16	PBT16	1.407	3.00 ± 1.32 ^bcdefg^
17	PBT17	0.435	2.00 ± 0.50 ^gh^
18	PBT18	0.863	2.50 ± 0.50 ^efgh^
19	PBT19	1.245	2.93 ± 0.21 ^cdefg^
20	PBT20	1.314	2.43 ± 0.12 ^efgh^
21	PBT21	1.06	3.40 ± 0.53 ^abcdef^

A DMRT test was used to check the significance level among the value. Values in a column with different lowercase letters are significantly different; (*p* ≤ 0.00001). CD value: 1.146 and SE(d): 0.566.

**Table 3:** Identified species of *Trichoderma*.

Isolate	Identified Species	Total Score	Query Cover (%)	Identity (%)	Accession No.	Genbank Accession No.
PBT3	*T. virens*	967	100	100	MN452840.1	0N678281
PBT4	*T. asperellum*	1024	100	100	MN046976.1	PP256386
PBT13	*T. harzianum*	1064	100	100	MF87546.1	PP256488
PBT9	*T. lixii*	1064	100	100	MK288146.1	PP256388

**Table 4:** GC-MS compounds of *Trichoderma* spp. and their chemical nature.

*Trichoderma harzianum*						
Sr. No.	Name of Compound	Molecular Formula	Molecular Weight	RT (min)	Peak Area%	Specific Role
1.	Nonanal	C_9_H_18_O	142	8.731	0.85	Antifungal and antibacterial
2.	Acetic acid, 2-ethylhexyl ester	C_10_H_20_O_2_	172	9.406	6.29	-
3.	Nonanoic acid	C_9_H_18_O_2_	158	11.249	0.71	-
4.	Benzeneethanol, 4-hydroxy-	C_8_H_10_O_2_	138	13.500	0.53	Antifungal activity
5.	2,4-Di-tert-butylphenol	C_14_H_22_O	206	14.563	0.58	-
6.	aR-Turmerone	C_15_H_20_O	216	16.495	1.65	-
7.	Tetradecanoic acid	C_14_H_28_O_2_	228	17.513	5.47	-
8.	Hexadecanoic acid, methyl ester	C_17_H_34_O_2_	270	19.243	5.00	Antifungal activity
9.	n-Hexadecanoic acid	C_16_H_32_O_2_	256	19.626	6.80	Antifungal activity
10.	6-Octadecenoic acid, methyl ester, (Z)-	C_19_H_36_O_2_	296	20.957	6.19	Antifungal activity
11.	1-Hydroxy-3-methylanthraquinone	C_15_H_10_O_3_	238	22.201	12.24	-
12.	Hexadecanoic acid, 2-hydroxy-1-(hydroxymethyl)ethyl ester	C_19_H_38_O_4_	330	24.509	2.95	-
13.	Bis(2-ethylhexyl) phthalate	C_24_H_38_O_4_	390	24.680	5.42	Antimicrobial activity, Antibacterial activity
14.	Octadecanoic acid, 2,3-dihydroxypropyl ester	C_21_H_42_O_4_	358	26.095	2.36	Antifungal activity
15.	Tetrapentacontane	C_54_H_110_	758	27.372	1.90	-
***Trichoderma lixii***						
**Sr No.**	**Name of compound**	**Molecular formula**	**Molecular weight**	**RT (min)**	**Peak** **area%**	**Specific area**
1.	Butanoic acid, 3-hydroxy-	C_4_H_8_O_3_	104	5.966	1.49	Antibacterial activity
2.	Acetic acid, 2-ethylhexyl ester	C_10_H_20_O_2_	172	9.408	4.61	-
3.	Benzaldehyde, 4-nitro-	C_7_H_5_NO_3_	151	12.409	1.59	-
4.	Dehydroacetic acid	C_8_H_8_O_4_	168	12.704	0.58	Antimicrobial activity
5.	3H-Pyrazol-3-one, 1,2-dihydro-5-methyl-	C_4_H_6_N_2_O	98	14.535	0.57	-
6.	2,4-Di-tert-butylphenol	C_14_H_22_O	206	14.564	0.60	-
7.	Tetradecanoic acid	C_14_H_28_O_2_	238	17.518	4.54	Antibacterial activity
8.	7,9-Di-tert-butyl-1-oxaspiro(4,5)deca-6,9-diene-2,8-dione	C_17_H_24_O_3_	276	19.114	1.52	-
9.	1-Hydroxy-3-methylanthraquinone	C_15_H_10_O_3_	238	22.196	1.38	Antimicrobial activity
10.	Bicyclo[3.3.0]octan-3-one, 6-iodo-2-methyl-, cis	C_9_H_13_IO	264	22.489	1.41	-
11.	N,N-Dimethyl-6-[4-methylphenyl]-1,2,4,5-tetrazin-2-amine	C_11_H_13_N_5_	215	23.569	2.48	-
12.	Bis(2-ethylhexyl) phthalate	C_24_H_38_O_4_	390	24.685	2.31	Antifungal activity
13.	Chloramphenicol	C_11_H_12_Cl_2_N_2_O_5_	312	26.198	42.16	-

## Data Availability

The original contributions presented in the study are included in the article, further inquiries can be directed to the corresponding authors.
